# Unusual Animal Behavior Preceding the 2011 Earthquake off the Pacific Coast of Tohoku, Japan: A Way to Predict the Approach of Large Earthquakes

**DOI:** 10.3390/ani4020131

**Published:** 2014-04-03

**Authors:** Hiroyuki Yamauchi, Hidehiko Uchiyama, Nobuyo Ohtani, Mitsuaki Ohta

**Affiliations:** 1 Department of Animal Science and Biotechnology, Azabu University Graduate School of Veterinary Science, 1-17-71 Fuchinobe, Chuo-ku, Sagamihara, Kanagawa, 252-5201, Japan; E-Mail: hiroyuki.yamauchi19@gmail.com; 2 Department of Human and Animal-Plant Relationships, Tokyo University of Agriculture, 1737 Funako, Atsugi, Kanagawa 243-0034, Japan; E-Mail: h3uchiya@nodai.ac.jp; 3 Department of Animal Science and Biotechnology, Azabu University School of Veterinary Science, 1-17-71 Fuchinobe, Chuo-ku, Sagamihara, Kanagawa, 252-5201, Japan; E-Mail: mohta@azabu-u.ac.jp

**Keywords:** pets, dairy cows, earthquake precursors, unusual behaviors, milk yields

## Abstract

**Simple Summary:**

Large earthquakes (EQs) cause severe damage to property and people. They occur abruptly, and it is difficult to predict their time, location, and magnitude. However, there are reports of abnormal changes occurring in various natural systems prior to EQs. Unusual animal behaviors (UABs) are important phenomena. These UABs could be useful for predicting EQs, although their reliability has remained uncertain yet. We report on changes in particular animal species preceding a large EQ to improve the research on predicting EQs.

**Abstract:**

Unusual animal behaviors (UABs) have been observed before large earthquakes (EQs), however, their mechanisms are unclear. While information on UABs has been gathered after many EQs, few studies have focused on the ratio of emerged UABs or specific behaviors prior to EQs. On 11 March 2011, an EQ (Mw 9.0) occurred in Japan, which took about twenty thousand lives together with missing and killed persons. We surveyed UABs of pets preceding this EQ using a questionnaire. Additionally, we explored whether dairy cow milk yields varied before this EQ in particular locations. In the results, 236 of 1,259 dog owners and 115 of 703 cat owners observed UABs in their pets, with restless behavior being the most prominent change in both species. Most UABs occurred within one day of the EQ. The UABs showed a precursory relationship with epicentral distance. Interestingly, cow milk yields in a milking facility within 340 km of the epicenter decreased significantly about one week before the EQ. However, cows in facilities farther away showed no significant decreases. Since both the pets’ behavior and the dairy cows’ milk yields were affected prior to the EQ, with careful observation they could contribute to EQ predictions.

## 1. Introduction

Many countries including, most recently, Japan, have suffered extensive damage due to earthquakes (EQs). On 11 March 2011, the EQ (Mw 9.0) occurred in Japan, which took about twenty thousand lives together with missing and killed persons. There are several studies on the short-term prediction of EQ occurrence [1,2,3]. Those studies have mainly focused on pre-seismic unusual physical and/or chemical variations near the epicenters, such as electromagnetic signals, ionospheric propagations, and radon gasses, emerging prior to EQs [1,2,3]. These anomalies have often been observed within a few weeks before EQs. 

Additionally, posteriori surveys concerning anomalous phenomena have been conducted. Macroscopic anomalies include unusual animal behaviors (UABs), abnormal sounds [4], EQ lights [5], EQ clouds [6], ground deformation [4], and abnormalities in the ground water [7]. Wadatsumi [8] investigated macroscopic anomalies preceding the Kobe EQ in Japan, on 17 January 1995, and found that the anomalous phenomena associated with animals constituted more than half of the total reported (872/1519). A similar ratio of UABs to the total macroscopic anomalies was reported after another EQ [9]. Many UABs had been observed within a week before the EQs [10]. The locomotive activities of mice drastically increased one day prior to the Kobe EQ [11], while the circadian rhythm of mice locomotion disappeared in the days before the Wenchuan EQ in China (12 May 2008, M = 8.0) [12]. Rikitake [13] noted that small animals and insects showed UABs first and then larger animals (birds, rodents and mammals) up to the hour before the EQ. 

The UABs of dogs and cats were observed most frequently within the 24 hours prior to the Kobe EQ [8]. These behaviors included “barking loudly”, “being panicked”, or “biting owners” in dogs, and “hiding”, “being restless”, “meowing pathetically”, “taking the kitten outside”, “climbing a high tree”, or “disappearing” in cats [14]. 

The hearing range is 67 to 44,000 Hz in dogs, 55 to 79,000 Hz in cats, and 31 to 17,000 Hz in humans [15]. Thus, dogs and cats can hear ultrasounds that humans cannot. The number of smell receptors, olfactory cells, of dogs and cats are over an order of magnitude more numerous when compared with the 12 million of humans [16]. The olfactory bulb contains approximately 280 million cells in dogs, 67 million in cats, and 5 to 20 million in humans [17,18]. Humans can detect odor concentrations from 10^−4.5^ molar (M) to 10^−5.0^ M. Remarkably, dogs can detect a concentration of 10^−17^ M [19,20,21]. With these superior senses, dogs and cats show a greater sensitivity to small changes in smell and/or sound in their environment than humans. The occurrence of behaviors is incomplete without stimuli. For UABs prior to EQs, possible candidate stimuli include changes in atmospheric pressure, changes in gravity, ground deformation (ground uplift and tilt changes), acoustic signals and vibrations due to the generation of micro cracks, ground water level changes, and emanations of gases and chemical substances [22]. With their extraordinary sensory abilities, it is possible that animals sense and respond to such stimuli. Animals might show UABs as results of which they felt anxiety for EQ precursors as described by Lott *et al.* [23]. In addition, there are some considerable theoretical and experimental evidences that animals may be responding to some physical or chemical anomalies prior to EQs; (1) massive amounts of positive airborne ions which cause changes of the stress hormone concentration in animals and humans as described by Grant *et al.* [24], Freund [25], and Freund and Stolc [26]; (2) large amounts of toxic gasses such as the carbon monoxide, which is odorless but deadly [26,27]; (3) electromagnetic anomalies such as ultralow frequency and extremely low frequency field which cause physiological effects [22,26]. However, the mechanism by which these stimuli are sensed remains undetermined. Another controversial point is the anecdotal and retrospective nature of the reports regarding UABs prior to EQs. At present; however, the best course of action is to collect as much information on unusual physical and biological phenomena before EQs as possible. 

The milk yield of dairy cows, although it is not a behavior, could be useful as an EQ predictor because it is often measured daily by instruments in the animal industry. In addition, milk yields are decreased by various stressors [28,29]. There are also reports that cows showed UABs prior to EQs [30,31]. If cows feel unusual physical or chemical variations prior to EQs, then milk yields could decrease. It may help to reveal the relationship between the seismic activities and UABs prior to EQs. The information on UABs prior to EQs could assist in predicting the next EQ occurrence. The aim of this study is to categorize the information on UABs prior to the massive 2011 Tohoku EQ in Japan and analyze the relationship between the information and seismic characteristics.

## 2. Material and Methods

### 2.1. Seismic Data

**Figure 1:**
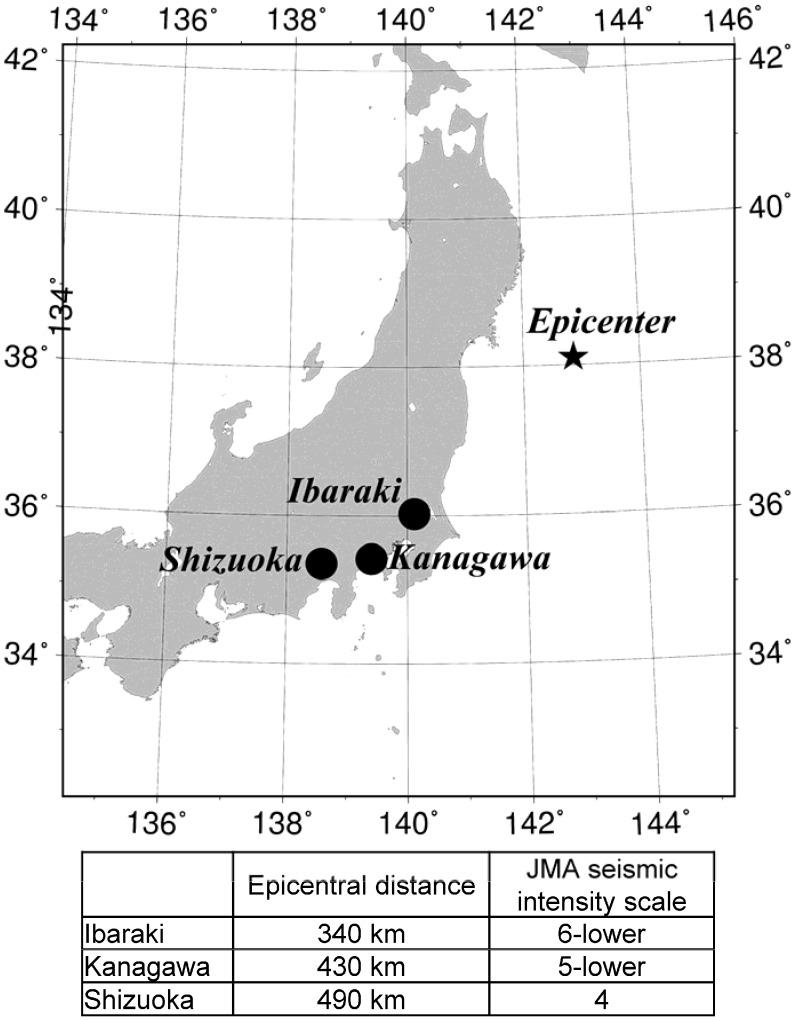
The epicenter of the 2011 earthquake off the Pacific coast of Tohoku, Japan. Black circles represent the approximate locations of three institutes with dairy cow milking facilities used in this study. The lower table shows the distances from the earthquake’s epicenter and approximate intensity on the Japan Meteorological Agency (JMA) seismic intensity scale of the three institutes.

On 11 March 2011, at 14:46 JST (05:46 UTC), a megathrust EQ with Mw 9.0 occurred in the Northwestern Pacific Ocean (138.104°N, 142.861°E) at a shallow depth of 24 km, which is formally named as “the 2011 off the Pacific coast of Tohoku Earthquake” (Tohoku EQ, Figure 1). This EQ occurred on or near the subduction zone plate boundary between the Pacific and North America plates. There was a foreshock of Mw 7.3 on 9 March 2011, at 11:45 JST (02:45 UTC), two days before the main shock.

### 2.2. UABs of Pets

The web survey, in association with Iris Ohyama Inc. (Miyagi, Japan), collected a large amount of data from across the nation from 6 December 2011, to 19 January 2012. The survey’s campaign website was entitled “The survey associated with the EQ and the pet” (managed by Iris Ohyama Inc.) and was limited to registered members of the pets’ informational-website of Iris Ohyama Inc., that had dogs and/or cats. The questionnaire contained three sections: general information about the pets, their living environments, and the UABs. The general information consisted of species (dog or cat), sex, gonadal status, and age. The questions about the living environment included the owner’s address and where the pets had been reared. To protect personal information, only the post code of the owner’s address was used. This still enabled us to analyze the relationship between the distance from the epicenter and the UABs. The question regarding the pet’s place of rearing had four options: “only indoor”, “mainly indoor”, “only outdoor”, and “mainly outdoor”. Respondents were asked whether they observed UABs preceding the Tohoku EQ in their owned pets. The questions relevant to UABs were composed of 15 behavioral types based on previous studies of UABs [8,14] and emotional responses (stress, anxiety, and fearfulness) [32,33,34]. The detailed contents of these behavioral types are shown in Table 1. This question allowed multiple answers. If the respondents had observed particular types of UABs, then they were asked when they were observed. This question consisted of six terms: “from a few seconds to minutes”, “from 1 to a few hours”, “1 day”, “from 2 to 3 days”, “from 4 to 5 days”, and “6 or more days” before the EQ. If respondents owned more than one pet, then they were asked about only the pet with whom they had the closest relationship. All procedures pertaining to personal information in this study were in accordance with the guidelines supplied by the Human Research Ethics Committee of Azabu University. 

**Table 1:** Types of unusual animal behaviors

	Abbreviations	Behavior types
1	Escaped	Animals escaped their normal home environment (home or yard)
2	Frightened	Animals were frightened by something
3	Barked (dog)	Dogs barked more than usual
4	Howled (dog)	Dogs howled more than usual
5	Vocalized (cat)	Cats vocalized more than usual
6	Restless	Animals were restless
7	Trembling	Animal’s bodies were trembling
8	Shaking off	Animals were shaking off a lot
9	Hiding	Animals were in hiding somewhere
10	Different place	Animals wanted to be in a different place than usual
11	No appetite	Animals had no appetite
12	Diarrhea	Animals had diarrhea
13	Vomited	Animals vomited
14	Stuck	Animals stuck close to the owner
15	Aggressive	Animals became aggressive
16	Other	Other

### 2.3. Milk Yields of Cows

With the aid of the institutes of animal industry in Kanagawa (Livestock Industry Technology Station; Kanagawa Agricultural Technology Center), in Shizuoka (Shizuoka Prefectural Research Institute of Animal Industry), and in Ibaraki Prefecture (National Agriculture and Food Research Organization) of Japan, we collected the daily milk yields of 86 Holstein cows from 1 January 2011, to 31 March 2011 (Figure 1). The population of each milking facility, which is a typical research center of the livestock industry in Japan, was 20 to 41 animals. The milking process was similar in all the facilities, with the cows being induced to the milking parlor and then milked by machines. They were individually identified by tags, and milk yields were counted using electronic milk meters. The milking frequency was twice a day in all of the facilities. The data measured were transferred to computers, and we used the total milk yield per cow per day. These measures are taken every day, not just in connection with this study. Milk yields from dairy cows increase for approximately four to eight weeks postpartum, and gradually decrease thereafter, with lactation being complete by approximately 40 weeks. Thus, it was necessary to remove the known factors affecting milk yields, such as the number of days after calving and environmental temperature and humidity, to verify the relevance of the EQ. 

### 2.4. Statistical Analysis

The clear range of epicentral distance which animals show UABs is undetermined. Chen *et al.* [35] found out that there was surface displacement which covered entire Japan and agreed with the fault mechanism since about 80 days before the Tohoku EQ, by using the daily data from 1,243 Global Positioning System stations, although we do not know what triggers the UABs prior to EQs. Therefore, we used reports in all over Japan for analyses. The locations of respondents were defined as the center of their postcode area, which was established using latitude and longitude, and their distance from the EQ epicenter was calculated. The distance was transformed using a common logarithm as in earlier studies [36]. Logistic regression analyses using generalized linear models with a binomial error distribution and a logit link function (glm function in R Software version 2.15.0) were performed to analyze relationships between the distance from the epicenter and the presence of UABs according to each term. The presence or absence of UABs in each term was used as a dependent variable, and the distance from the epicenter was used as an independent variable. All analyses were conducted in dogs and cats separately. 

We removed the effects of known factors that decrease milk yield before the analysis. First, we removed the effect of the number of days after calving by using WOOD’s lactation curve model [37]. Second, we removed the effect of the temperature-humidity index [38], which is used as the index of heat stress for cows [39], by using two-phased regression model. Third, final variations in milk yield were calculated by subtracting the average milk yield after removal of the factors one to seven days before the EQ from those of the current day, and these final variations were used for the next analysis. We used milk yields from the 1 to 31 January 2011, as the basal period, assuming that any EQ-related variation had not begun. Then, the differences between milk yields in the basal period and those in each day, from the 1 February to the 11 March 2011, were analyzed using a repeated measures two-way ANOVA and *post hoc* test. If there was an interaction between the milking facility and the days, then the differences between the milk yield in the basal period and those in each day were analyzed using a repeated measures one-way ANOVA and *post hoc* test for that facility. All statistical procedures were analyzed by using R Software version 2.15.0. The cutoff for statistical significance in all tests was established as a *P* ≤ 0.05.

## 3. Results

### 3.1. UABs of Pets

A total of 1,976 responses were collected in this study. Fourteen responses were omitted, one from abroad, and 13 that lacked reliability and/or contained contradictory information. Of the 1,962 usable responses, 1,259 were dog owners and 703 cat owners. The details of the general information on the pets and their living environments are shown in Table 2. The minimum and maximum distances of the dog owners from the epicenter were 140 km and 2,350 km, respectively. For cat owners, they were 140 km and 1,950 km, respectively. 

**Table 2:** General characteristics of the pets.

	Dogs	Cats
***n***	1259	703
**Sex**		
Male (intact)	665 (329)	373 (57)
Female (intact)	589 (235)	327 (41)
Unknown	5	3
**Age (years old)**		
<1	69	34
1–3	372	249
4–6	303	144
7–9	261	88
10–12	163	76
13–15	63	44
>16	13	46
Unknown	15	22
**Rearing place**		
Indoor	1159	677
Outdoor	100	26
**Distance from epicenter (km; mean ± S.E.)**	527.5 ± 7.8	498.7 ± 9.7

**Figure 2:**
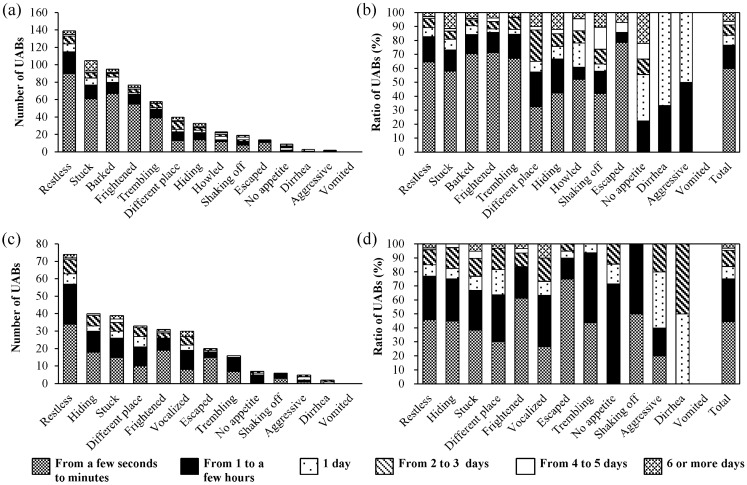
(**a**) Numbers of unusual animal behaviors (UABs) of dogs in each behavioral category and the time of occurrence prior to the 2011 earthquake off the Pacific coast of Tohoku, Japan; (**b**) Relative ratio normalized as % of the total; (**c**) Numbers of unusual animal behaviors (UABs) of cats in each behavioral category and the time of occurrence prior to the 2011 earthquake off the Pacific coast of Tohoku, Japan; (**d**) Relative ratio normalized as % of the total.

A total of 236 (18.7%) dog owners and 115 (16.4%) cat owners observed UABs of their pets (Figure 2). The numbers of dog and cat owners that observed a single UAB were 55 and 39, respectively, and the numbers that observed more than one UAB were 181 and 76, respectively. In observed UABs, restless behavior was the most common, whereas diarrhea, vomited, no appetite, and aggressive behavior occurred only occasionally in dogs and cats. The timing of the UABs was different between dogs and cats. In dogs, 60.0% of the UABs occurred “from a few seconds to minutes” before the EQ. The distribution of the remaining UABs was as follows: 16.7% were “from 1 to a few hours”, 7.1% were “1 day”, 7.3% were “from 2 to 3 days”, 2.6% were “from 4 to 5 days”, and 6.3% were “6 or more days” before the EQ. In cats 44.6% of the UABs occurred “from a few seconds to minutes” and 30.4% occurred “from 1 to a few hours” before the EQ. The distribution of the remaining UABs was as follows: 9.0% were “1 day”, 11.5% were “from 2 to 3 days”, 1.6% were “from 4 to 5 days”, and 2.9% were “6 or more days” before the EQ. 

Results of a regression analysis of each term are shown in Table 3 and Table 4. Dog UABs in the categories “from a few seconds to minutes (*P* < 0.001)”, “from 1 to a few hours (*P* = 0.020)”, “1 day (*P* = 0.019)”, and “6 or more days (*P* < 0.001)” before EQ were significantly closer to the epicenter. Also, cats UABs at 2 to 3 days (*P* = 0.014) increased as the distance to the epicenter decreased. 

**Table 3:** Results of regression model to evaluate relationships between the epicentral distance and the presence of unusual behaviors of dogs during each time period; CI = confidence interval *b* = coefficient.

	*b*	*P* value	Odds ratio	95% CI	
				lower	upper
From a few seconds to minutes	−2.021	<0.001	0.149	0.067	0.326
From 1 to a few hours	−1.605	0.020	0.225	0.062	0.824
1 day	−2.335	0.019	0.130	0.020	0.836
From 2 to 3 days	−1.633	0.106	0.190	0.029	1.277
From 4 to 5 days	1.356	0.456	3.742	0.116	139.779
6 or more days	−4.408	<0.001	0.014	0.001	0.128

**Table 4:** Results of regression model to evaluate relationships between the epicentral distance and the presence of unusual behaviors of cats during each time period; CI = confidence interval *b* = coefficient.

	*b*	*P* value	Odds ratio	95% CI	
				lower	upper
From a few seconds to minutes	−0.848	0.155	0.428	0.132	1.373
From 1 to a few hours	−1.281	0.071	0.278	0.068	1.111
1 day	1.298	0.308	3.664	0.307	46.573
From 2 to 3 days	−2.974	0.014	0.051	0.004	0.525
From 4 to 5 days	−1.082	0.666	0.339	0.002	47.806
6 or more days	0.174	0.905	1.190	0.069	21.144

### 3.2. Milk Yields of Cows

The institutes of animal industry in Shizuoka, Kanagawa, and Ibaraki Prefecture of Japan are located at 490 km, 430 km, and 340 km, respectively, from the EQ’s epicenter (Figure 1). Table 5 is a list of EQs that satisfied the Dobrovolsky radius condition [40] recorded at least one of the three local institutes from 1 February 2011 to 11 March 2011 (14:46 JST). 

**Table 5:** A list of earthquakes that satisfied the Dobrovolsky radius condition recorded at least one of the three local institutes from 1 February 2011, to 11 March 2011.

Date	Time	Distance from epicenters (km)			Dobrovolsky radius (km)	Depth (km)	Magnitude
		Ibaraki	Kanagawa	Shizuoka			
05/02/2011	10:56	140	130	190	170	64	5.2
10/02/2011	22:03	170	260	320	210	48	5.4
24/02/2011	14:36	30	110	170	40	73	3.6
26/02/2011	04:12	180	140	190	140	56	5.0
27/02/2011	02:18	240	190	140	140	4	5.0
27/02/2011	05:38	240	200	140	230	4	5.5
07/03/2011	15:13	90	30	70	40	140	3.8
09/03/2011	11:45	380	470	530	1380	8	7.3
09/03/2011	11:57	390	480	540	460	12	6.2
09/03/2011	11:58	370	460	520	380	21	6.0
09/03/2011	13:36	400	490	550	420	11	6.1
10/03/2011	03:16	350	440	500	570	29	6.4
10/03/2011	03:44	400	490	560	510	36	6.3
10/03/2011	06:22	360	460	520	510	18	6.3
10/03/2011	06:23	350	450	510	840	9	6.8
11/03/2011	14:46	430	340	490	7410	24	9.0

**Figure 3:**
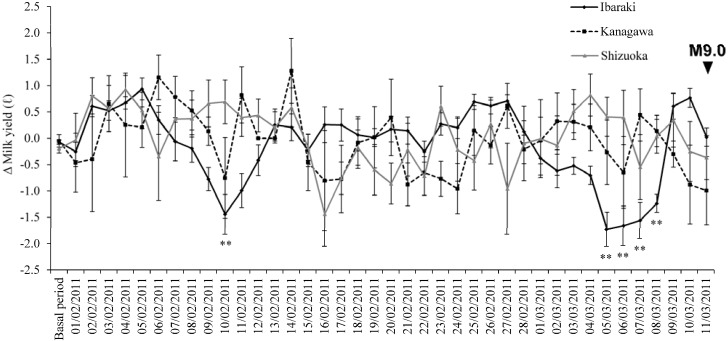
Changes in the dairy cow milk yields (mean ± S.E.) modified by days after calving and environmental factors at three institutes in Japan from 1 January 2011, to 11 March 2011. Darkened triangle (▼) indicates the occurrence of the earthquake. A double asterisks denote a significant difference at P < 0.001 compared with milk yields from 1 January 2011, to 31 January 2011, at the same institute.

Milk yields analyzed for this period showed a normal distribution. Standard deviations for milk yields at the three institutes from 1 January 2011, to 11 March 2011, were 0.621 at Ibaraki, 0.639 at Kanagawa and 0.601 at Shizuoka. Milk yields varied among the days (repeated measures two-way ANOVA, *F* = 1.730, *P* = 0.003), although the main effect among the facilities was not significant (Figure 3). An interaction between the days and the milking facilities was found (*F* = 1.778, *P* < 0.001). The result of a repeated measures one-way ANOVA analyzing the difference between milk yield during the basal period and during each day prior to the EQ at each institute revealed that only the Ibaraki Prefecture facility varied among the days (*F* = 8.875, *P* < 0.001). *Post hoc* tests indicated that milk yields on 10 February 2011 (*P* < 0.001), 5 March 2011 (*P* < 0.001), 6 March 2011 (*P* < 0.001), 7 March 2011 (*P* < 0.001), and 8 March 2011 (*P* = 0.001), were significantly decreased in comparison with the basal period in Ibaraki (Figure 3). 

## 4. Discussion

The clear findings of this survey were that UABs of pets and decreased milk yields from cows occurred about one week before the EQ. Most of the UABs of dogs and cats were observed within one day before the EQ, and the decreased milk yield occurred in Ibaraki Prefecture, 340 km from the EQ’s epicenter.

The numbers of reports in which pet owners observed UABs of their pets were 18.6% for dogs and 16.6% for cats. The most frequently observed UAB in dogs and cats was restless behavior and “stuck close to the owner” was also indicated as a UAB in both species. Cats were described as “hiding” or “escaped” more often than dogs; however, this might be due to the natural difference between the species’ behaviors in which cats are more able to act three-dimensionally and go through narrower interspaces than dogs [41]. Although the options of diarrhea, vomited, and no appetite were used for visually apparent behavioral changes, they were rarely observed in either animal. 

The ratio of total UABs increased in both animals as the time of the EQ occurrence neared. Many UABs prior to the Christchurch EQ in New Zealand (4 September 2010, Mw = 7.1) occurred within one hour of the EQ [42]. Although the exact time of the UABs were not asked for in this study, the variation in the UABs occurrence with time seemed similar to that of the Christchurch EQ. UABs were observed from a few seconds to minutes before the EQ, which could include responses to P-waves as described by Kirschvink [43]. In the Tohoku EQ, P-waves arrived at the closest area from the epicenter about 20 seconds before the arrival S-waves [44]. Thus, there was sufficient time to notice the behavioral changes of pets by their owners. In fact, three out of 84 respondents replied that “behavioral changes were shown before EQ sounds”. This suggests the presence of acoustic stimuli that could be discerned even by humans. No seismic swarms, as foreshocks of the Tohoku EQ, were observed before 9 March 2011; however, a number of large and small EQs occurred from 9 March 2011 (11:45 JST) to the Tohoku EQ on 11 March 2011 (14:46 JST), including 56 EQs of more than 3.5 M and 18 EQs of more than 5.0 M. Furthermore, 18 EQs satisfied the Dobrovolsky radius condition in the area nearest the epicenter. The UABs of dogs and cats after 9 March 2011, might include stress responses to shaking caused by these EQs. Therefore, the real ratio of UABs as precursor of the Tohoku EQ were observed after 9 March 2011, might be lower than indicated by the results of our survey. However, we consider that the UABs of pets occurred mostly in the few hours preceding the largest EQ (Mw = 9.0). 

The UABs of dogs increased as their distance from the epicenter decreased, except from two to three days and from four to five days before the EQ, whereas cats showed the same correlation from only two to three days before the EQ. Previous research found the same relationship between the number of UABs and the distance from the epicenter for many EQs [4,36]. UABs prior to EQs are considered as stress or emotional responses to physical or chemical variations. Some physical or chemical variations prior to the Tohoku EQ emerged during various time periods, and the emerged anomalies did not necessarily remain until the EQ occurred [45,46,47]. The existence or non-existence of a relationship between UABs and the epicentral distance depended on precursory terms. These irregularities of physical or chemical states might cause the UABs identified in this study, although we do not know what triggers the UABs prior to EQs. However, significant relationships between epicentral distance and UABs in dogs “1 day”, “from 1 to a few hours” and “from a few seconds to minutes” before the Tohoku EQ, and those in cats “from 2 to 3 days” before the Tohoku EQ might include effects from stress responses to shaking caused by foreshocks after 9 March 2011. 

There are some suggestions for possible stimuli, which cause UABs prior to EQs [22,24,26]. For example, Freund and Stolc have suggested that animals might respond to massive amounts of positive airborne ions, massive amounts of toxic gases and electromagnetic waves of ultralow and extremely low frequency [26]; however, the clear mechanism for UABs has remained unknown. At present time, to examine whether UABs are useful in EQ predictions, longitudinal observations of UABs are important and essential. It may be an effective prediction method since the objective behavior is observed everyday automatically. In fact, changes in the locomotive activities of mice before large EQs were reported by Yokoi *et al.* [11] and Li *et al.* [12]. Lott *et al.* [48] reported that ratios of UABs prior to EQs differed between some events, even if these occurred with similar distance, depth and magnitude. Longitudinal observations could enable to know that animals show UABs more frequently prior to what kind of EQs. In this study, restless behavior was the most frequently observed UAB in dogs and cats. It may be useful to quantify the frequency of this behavior. However, this result is based on only one case from an extremely large EQ, so further studies are required. 

The milk yield from milking facilities at institutes in Shizuoka and Kanagawa Prefecture were not significantly affected by the coming EQ, but the facility in Ibaraki showed lowered milk production 6 days before the EQ. The decrease in the milk yield continued for four days. This might be because Ibaraki was the closest of the three institutes to the epicenter. If so, milk yield might be useful as an EQ precursor. Furthermore, these decreases of milk yields were probably not caused by fear responses to the EQ’s shaking, because no seismic swarms that satisfied the Dobrovolsky radius condition [40] occurred near the location of the institute in Ibaraki Prefecture from the 5 to 8 March 2011. The 7.3 M EQ on 9 March 2011, did not influence milk yields, because there were not significant decreases after 9 March 2011, at the three institutes. Milk yields on 10 February 2011, also decreased compared with the basal period. This decrease might have been a precursor for the EQ (M = 5.4) that occurred the same day, although the possibility that this decrease was a precursor of the Tohoku EQ cannot be excluded. As the milking work was already finished at the time of this EQ (22:03 JST), the decrease was not a response to the shaking. However, milk yields before other EQs that satisfied the Dobrovolsky radius condition did not show significant decreases. Tests for relationships between milk yields and more EQs using a longer data period could reveal differences between EQs that affect milk yields and those that do not. 

Our study revealed the time periods in which the UABs of pets and changes in the milk yields of dairy cows occurred. About 80% of pet UABs were observed within one day of the EQ, whereas cow milk yields decreased six days before the EQ, which preceded the UABs of dogs and cats. Milk yields in each individual have been measured by many institutes in the animal husbandry industry to manage and improve productivity, and the UABs of pets have been observed directly by owners. These two phenomena could contribute to EQ predictions if people carefully observe whether pets show UABs, especially restless behavior, and when dairy cow milk yields decrease, as EQ precursors. 

## 5. Conclusions

Our study revealed the characteristics of pet UABs and of changes in the milk yield of dairy cows associated with an extremely large EQ. The number of UABs decreased as the distance from the epicenter increased, as seen in previous research. The results suggest that the restless behavior is the most common UAB prior to EQs. Milk yield decreased from four to six days before the Tohoku EQ in the facility closest from the epicenter (340 km). Our study indicates that these two phenomena could contribute to the prediction of EQs, but the longitudinal and objective measurement of UABs and the verification of the relevance between milk yield levels and other EQs need to be performed. 
